# Cryptic Bacteriemias

**Published:** 1916-06

**Authors:** E. C. Thrash

**Affiliations:** Atlanta, Ga.


### CRYPTIC BACTERIEMIAS.

By E. C. Thrash, M.D., Atlanta, Ga.

The infections which I shall describe under this head are those, the causative factors of which enter the circulatory system through unknown or unsuspected portals, such as previously existing traumatisms, broken or diseased mucosae of the digestive and respiratory tracts, especially around the teeth and in the fauces, previous septicemias that had been considered well, such as those arising from abortions, parturitions, acute middle and internal ear disturbances, infected injuries to the skin, together with all rheumatic and rheumatoid infections. These cryptic bacteriemias produce the injuries upon the tissues in a quiet insidious way, long after the areas through which they enter have healed, and the acute sepses which may have followed become apparently well, often without the clinician associating them with the initial disturbance. They are much more common than one who does not look for them might think, and when sought for properly are found to be the underlying causes of many affections which had been attributed to other causes.

From the standpoint of their behavior, we divide bacteriemias into two classes,—those, the germs of which are nonpyogenic and produce granulomatous disturbances, and those caused by germs which are more or less pyogenic and produce ordinary inflammatory processes. The most common germs producing granulomatous diseases are the tubercle bacillus, siprochaete and leprosy bacillus. These have all been carefully studied under the respective diseases which they produce, and since the discussion of them would lead us too far afield and make the subject-matter too voluminous and variable, they will be dismissed without further comment.

The pyogenic types are the ones which should arouse our interest, since they lead us into fallow grounds that have been as yet almost wholly uncultivated, and offer fruitful fields for the clinician and pathologist. The germs usually found are the streptococcus, staphylococcus, pneumococcus and typhoid bacillus in all their varying strains.

There are but few local infections in which the bacteria producing them do not reach the circulatory system. These systemic infections, however, vary all the way from types that are so slight that they are imperceptible and unrecognized, to those that are sufficiently virulent to produce death from acute bacteriemia and septicemia in only a few days. The usual course in acute bacteriemia is either for the system to be overwhelmed and death to ensue promptly, or for the antibodies with other antagonists to the invaders, to overcome and eradicate them completely, leaving the individual well, except the destructive changes that had been produced by the acute invasion. There are many exceptions to this course, however, and the purpose of this essay is to call attention to these. In such exceptional instances, there is after a variable length of time an abatement of activities of the invading germs, and the somatic cell structures become more or less indifferent to these enfeebled activities, resulting in a smouldering low type of infection, characterizing itself by innumerable forms of disturbances, varying in accordance with the kinds of tissue attacked, the resistance of the host, the type of the germ that is making the attack, and its degree of virulence.

Nature's several known modes of defense against pathogenic bacteria are: Walling them in by round cell infiltration, the elaboration of antibodies for disintegrating them and precipitating their metabolic products, by phagocytosis, and by taking them into lymph spaces where she attempts to destroy them in the lymphatic gland system. All these processes aid in the production of the pathology of inflammation. Phagocytosis, however, takes a more important part in producing pathology than is usually supposed, as this process is thought of as being confined to the white blood cells. It is accomplished not only by these, but by the fibroblasts developed from the fibrous tissue in the process of repair, and epithelioid cells from the endothelium of both the blood and lymphatic circulatory system. The endothelial cells of lymph spaces are included in the latter, and since the serous lined cavities of the joints, abdomen and pleura are modified lymph spaces their cells also are included. This explanation is to prepare us better for what follows.

If, after a period of time, nature fails to make a complete destruction of the germs which are circulating in the blood, she declares a truce toward them. There is still, though, a more or less feeble conflict between the host and invader in which a sufficient toxicity is produced to make the former cognizant of the fact that it still has an enemy within its gates. This conflict is so obscure and insidious that the pathologist, unless he searches quite carefully and along proper lines will only find the tissue changes produced by this somatic cell struggle, and will not discover the offender or the underlying cause of the pathology.

In discussing the changes produced by cryptic bacteriemias, it is necessary to place them in three groups: Those which take on a more or less acute course, those that are sub-acute, and the more chronic forms. The active forms keep the clinician constantly appraised of their existence, and where the resistance of the individual is only slight, and especially if he be a child, the germs, on account of agglutinating and precipitating factors in the processes of immunity become clumped so that they can be better attacked by bacteriolysis. This process, like many others of nature's ordinarily beneficient ones, proves sometimes pernicious, and these clumps of bacteria become lodged in the constricted capillaries of the bony structures, usually about the epiphyses, where there is terminal circulation plugging them, and producing microscopic infarcts, thus furnishing pabulum in which the germs can take on new life, and an inflammatory process is established which results in osteitis, periostitis and osteomyelitis, all so familiar to the surgeon. However, if the germ is less inclined to be pyogenic, as the streptococcus, it may confine its activities to the serous linings of the channels in which it is circulating, thereby producing endocarditis, endarteritis, lymphadenitis, arthritis, rheumatism and various rheumatoid infections. This type often produces direful ravages upon the valves of the heart.

The less acute forms may be almost imperceptible, producing only a feeling of malaise with occasional slight temperature and objective symptoms just sufficient to show that the patient is in bad health. The cause of this slight illness can not be revealed except by a blood culture, which, if correctly done, will show the offending agent. Nature is constantly struggling to checkmate this enemy, and in so doing she finally gets it so subjugated that all activities may become quiescent until she is caught off her guard by a lowering of the vital forces. Then the germ after months or years may again begin to make inroads upon the tissues. When this takes place the host often brings new weapons to bear in the form of previously mentioned endothelial cells which line the circulatory system, or synovial membranes.

The normal shape of an endothelial cell is flat because its function is to line cavities and tubes. When it becomes a phagocyte, however, it changes from a flat to a round cell. This is why capillary vessels disappear in acute localized inflammatory areas. Their flattened cells round up and become soldiers, thereby disintegrating the vessels. In chronic conditions, where there is no localized inflammatory process in capillary areas, and where the bacteria are being constantly whipped around in the blood stream, the only opportunity these endothelial cells have of attacking the invading enemy is to catch it up as it flows through the vascular system. Therefore, on account of the stimulus produced by the infection, the endothelial cells round up, and project out into the lumen of the vessel which they help to form, for the purpose of attacking the passing germs. The bacteria floating near, lodge against them and by agglutinating processes become attached. These cells in this way destroy them by phagocytosis. When this process becomes very active an inflammation and phlebitis results. The cells upon the roughened wall of the vessels not only attach the bacteria, but the structural elements of the blood also find lodgment here, resulting in a thrombus,—another one of nature's beneficent efforts which become prenicious. These thrombi furnish a medium in which their bacterial content may proliferate rapidly. The inflammation and coagulum progresses along the course of the veins, fragments of thrombotic structures often breaking away and finding lodgment in the lungs, setting up disturbances in the form of emboli and infarctions.

The chronic types are still more insidious in their activities than either of the other two. Many of the so-called auto-intoxications and chronic fibroses of the vascular system, kidneys and liver, result from the slow acting toxic products of these almost non-pathogenic germs that have acquired the habit of continuing to live in the fluids of the body on account of these fluids making only a feeble effort to resist them.

A common type of disturbance produced by this process, in addition to the ones above mentioned, is chronic rheumatoid arthritis. The serous coverings of the joints are invaded by bacteria just as they invade the lymph and blood channels and the former's feeble effort to checkmate the progress of this invasion is quite similar to the process previously described on the part of the veins, except that it is slower and less effectual. There is not only a building up of epitheloid cells from endothelium for phagocytic purposes, but there is a slow formation of fibroblasts in an unsuccessful effort at repair and to restore the disturbed tissues to normal. This results in a fibrosis similar to the one mentioned in connection with the vascular system and kidneys, except that the serous coverings of the joints are in such intimate contact with the bone that, when the fibroblastic structures lay down their deposits in the formation of fibrous tissue, by a process of metamorphosis they become bone cells developing progressively bony excresences, exostoses and nodular projections until the joint may finally become obliterated. These germs may also make slow inroads upon the heart valves until a fibrosis will take place in these structures to a degree that varying types of stenoses and other insufficiencies may result. It is not possible in so short a paper to follow the varied activities of these bacteria into all of their ramifications, or to mention all the phases under which they appear, or the pathological changes which they produce. One can at most only touch upon a few of them.

These bacteriemias can be diagnosed only by blood cultures, and the technique which I have found most efficient is as follows: Draw out from five to twenty cubic centimeters of blood into a sterile syringe from one of the veins of the arm, previously cleansing the arm with iodine. While the arm is still moist with iodine plunge the needle, the point of which has previously been dipped into iodine, into the vein. There are but few methods of technique in the handling of bacteria where there is greater possibility of contamination than in this one. To guard against this, one should, after drawing the needle out of the vein force out a few drops of blood, then draw the piston slightly back just enough to empty the needle, after placing a pledget of sterile cotton over its end to prevent drawing in bacteria from the air. Plunge the needle then into boiling water. This is to kill such germs as may lodge in the needle point as it is drawn out. Drawing the blood out of the needle is done in order that boiling will not congeal it, thereby plugging the needle so that the blood cannot be forced out of the syringe. Inoculate by slightly pulling to one side the plug of cotton stopping the receptacle containing the culture medium, and forcing the needle down through the cotton or between the cotton and the flask. Inject from five to ten cubic centimeters into each flask that is to be inoculated.

I have found the best method to get a growth is to use from twenty-five to one-hundred cubic centimeters of simple or glucose bouillon. The addition of blood in part supplies the bacteria with the substance in which they are accustomed to grow. I have never been able to get a growth upon solid medium with bloods thus infected. In some instances the germs grow well in the incubator, in others at room temperature, whereas some have to be grown anaerobically; therefore it is well to incubate them both at room and body temperature, aerobically and anaerobically. I find many infected bloods from which I can get a feeble growth sufficient to show bacteria are present, but from which, with all the teasing and manipulating possible I can not get a growth sufficient to make a vaccine. Contaminations can usually be detected, since they grow luxuriantly, filling the culture medium over night with germs usually of the staphylococcus variety. The mere fact, however, that they are staphylococci does not imply that they are extraneous, as this type probably is found in blood more frequently than any other. One should look with doubt upon the genuineness of germs which grow well upon the first inoculation. The haemic ones grow better after each transplanting, but at first they usually grow feebly.

The only specific measures for relieving these conditions is to establish an immunity. The best means for accomplishing this is in the use of vaccines. The result of vaccine therapy, however, has not been so brilliant in haemic infections as in others of a more localized nature. No doubt, the reason of this is because the dead bacteria and their metabolic products are injected subcutaneously and they reach the blood stream by such a circuitous route, and so slowly that they do not supply a suitable stimulus for building up the proper qualities and quantities of antibodies. Better results are obtained by injecting the vaccines directly into the blood stream. In adopting this procedure great care should be used to break up all bacterial clumps, observing the mechanical condition of the vaccine carefully while the count is being made to see that this is done, otherwise the clumps might produce small emboli in the lungs that would give the patient more or less discomfort.

